# Intestinal Worms—their Effects, &c., in the Adult

**Published:** 1848

**Authors:** W. Matthews

**Affiliations:** Putnam county, Indiana


### ARTICLE II.

*Intestinal Worms—their Effects, &c., in the Adult.* By W. Matthews, M.D., of Putnam county, Indiana.

Professor McLean, in his recent course of lectures in Rush Medical College, remarked the infrequency of intestinal worms in the human subject in the western country generally; but, from several years' observation, I am satisfied that this particular locality is an exception to the general rule. Here, intestinal worms—the ascaris lumbricoides to which I refer—are extremely common; indeed, not one child in three, I am very sure, arrives at the age of ten years without having suffered, more or less, from the morbid effects of invermination. Nor are adults exempt from these troublesome parasites. Within the last few years, numerous instances of verminous irritations in the adult have fallen under my observation.

An investigation into the causes which favor the more than usual generation of this species of worm—the lumbricoides—in particular localities and at particular times, might be a subject worthy a more extended notice than I can give it here. Some pathologists have, I believe, attempted to account for the almost universal prevalence of intestinal worms at certain seasons, and as confined to certain districts, by ascribing their *origin* to atmospheric influences. That atmospheric influences may *assist*, or act as a remote cause, in inducing that condition of the alimentary canal favorable to the production of worms, I have no doubt; but I imagine that this cause is, of itself, wholly inadequate to their evolution. Let intestinal worms originate how they may—whether from ova, or as many, I think rather fancifully, suppose, spontaneously, without a vital germ—all agree, I believe, that whatever deranges the prima via and induces indigestion, favors their production. Unwholesome, heavy, indigestible articles of diet too long continued, together with the malarial atmosphere under which we live, are the principal, perhaps only causes, which favor the origin and accumulation of worms in the human subject, in my opinion.

I may be allowed to state the following facts as confirmatory of this view, respecting the production of worms. A neighbor of mine, in the spring of the year 1846, lost, by death, a lot of five or six young hogs, under circumstances which led him to infer they had contracted a distemper, communicable by contagion. Happening to be present when one of the shoats died, I proceeded to open it immediately, and to my astonishment, was readily enabled to discover the cause of death, in an immense accumulation of the lumbricoides, in the upper portion of the jejunum and in the stomach. About eighteen inches of this intestine was completely distended with worms, about one hundred and fifty, of an average length of eight inches, being congregated thus together. The intestine was completely gangrenous.

Upon further observation, the whole number was found to have died from the same cause as that just stated.

Now, upon enquiry, I was enabled to ascertain that these young hogs, all the offspring of one litter, had been treated in the following manner: they had been confined to a large wood-land pasture, and white oak acorns, of which an abundance was upon the ground to which they were confined, constituted their sole food. These acorns, though in a putrescent condition, occasioned by the alternate freezing and thawing to which they were subjected, were sufficiently nutritious to keep them in a thriving condition; but, unwholesome as they must have been, they were, doubtless, the cause which gave rise to a condition favorable to the production of these parasitic creatures, the amazing increase in whose number ultimating in the death of the annimals, partly, perhaps, from obstruction in the bowels, and partly, no doubt, from the morbid irritation which they induced. Now there can be scarcely a doubt but that persons—children more particularly—so circumstanced as to be placed upon a diet, although sufficiently nutritious, whose qualities are not well adapted to the digestive function, will not unfrequently favor the evolution of worms in the digestive apparatus itself. Indeed, these aberations of digestion seem to constitute a climate, so to speak, every way favorable for the sprouting and rapid maturation of worms. These suggestions being correct, it will readily be seen how a family, or a whole community, may suffer from simultaneous invermination. They live alike, upon the same articles of diet, the same atmosphere surrounds them, and these circumstances are unfavorable to the performance of healthful digestion; and this last circumstance, it will be remembered, favors the generation of these troublesome accompaniaments of bad digestion.

Having premised these remarks, it remains for me to indicate some of the more obscure symptoms as not uncommonly met with in certain adult individuals, more especially those of the nervous temperament. And I cannot, perhaps, better illustrate these symptoms, than by narrating a few cases from my note book.

Case I.—Mrs. B., aged about 35, and mother of six children for several years previous to 1843, had suffered from often repeated hysterical paroxysms, many of which were of extreme violence. About this time I was called on to treat her. Gastro-intestinal irritation, with frequent fits of religious shouting, crying, &c., accompanied with irregular spasms, were the symptoms present. Stimulants of the anti-spasmodic class, purgatives, &c., were prescribed, but were almost immediately rejected by vomiting—the stomach being in an extremely irritable condition. She was ultimately blistered over the stomach, and it was only after the lapse of many days, that she partially regained her usual feeble health. These "fits" continued to recur, with increasing frequency, notwithstanding the remedies of a tonic nature prescribed for their prevention, until the fall of 1844, when I prescribed an anthelmintick, followed by a tonic plan of treatment. An immense number of lumbricoides were discharged ; the patient rapidly regained her health, and has, in general, enjoyed very good health ever since.

Case II.—Mrs. N., aged, perhaps, about twenty-five, and mother of three children, suffered from symptoms of indigestion, accompanied with nervous palpitations, great anxiety, and prostration, in the autumn of the year 1844. These symptoms became aggravated, and there was added to them *ardor urinæ*, together with what I shall style, for want of a more significant term, *nuralgia* of the uterus itself, of a very distressing character. The patient was a prostitute, and at first I suspected she had contracted a gonorrhœa ; but, satisfying myself that this was not the case, a prolapsus uteri was searched for, but not found to exist. These distressing symptoms, partially palliated by sedatives, with loss of appetite, emaciation, &c., continued to torment my patient for several weeks, notwithstanding the alteratives, cathartics, &c., with which she was being dosed all the while. At this stage of the disorder, I was, from some circumstance, led to suspect the existence of intestinal worms, and prescribed a vermifuge. This, in the language of the nurse, expelled "all but a chamber-pot full" of the round worm. My patient regained her health rapidly, and, soon afterward, became pregnant by one of her numerous lovers!

Case III.—Miss B., a prostitute also, was placed under my care for treatment in the autumn of 1846. The history of this case is so similar, in every particular, to the last, that it would be but repetition to give it. The *ardor urinæ*, the *nuralgia* of the uterus, and the nervous symptoms, were all present; and, after much perplexity on my part, were relieved by the same means.

Case IV.—Mr. R., about twenty-five years of age, of excellent constitution, scarcely ever having experienced a day's sickness, in the spring of last year, found his health gradually declining. He was feeble, appetite capricious, tongue coated, bowels irregular, costiveness, alternately with diarrhœa. I was consulted. He took some medicines for these symptoms, and supposed himself somewhat benefitted by them; but they recurred again and again, and were again and again abated by a temporizing plan of treatment, but without permanent advantage. The passage of a worm or two from his bowels, induced me to advise an active anthelmintic, which, bringing away a large number of the common round worms, afforded him complete relief from all unpleasant symptoms, up to this time.

I might narrate many other cases in all respects similar to those already given; but I hope enough has been said under this head to put the young practitioner on his guard as to the proper manner of relieving certain cases, which, if practising in this country, must, not very infrequently, come under his management. If he be observing, when hysterical persons—and especially those of suspicious morals—fall under his care, he will, in general, be able, although the attendant symptoms may call the attention of both patient and friends to sympathetic complications remote from the seat of the disorder, to diagnose the existence of verminous irritation, if it be present. The expulsion, by vomiting or from the bowels, of a single worm, should excite his suspicion; and as anthelmintics may be given in almost all conditions of the system, they should be prescribed—even on the empirical principle, if they do no good they will do no harm, for these are perplexing cases.

From what has been said, I would not be understood as contending that even a considerable number of hysterical complaints, as occurring in this vicinity, are the offspring of invermination; but my own observation warrants me in asserting that some of those obscure disorders, reckoned most commonly hysteria, are but the symptoms of verminous irritation; and, in a vast majority of instances, at least, intestinal worms date their existence *subsequently* to impaired digestion.

The treatment, then, of such cases as those above narrated, is at once manifest. The worms, though the *result* of primary disorder themselves, constitute a first indication in the treatment. A second indication is to restore to healthful play the digestive system; and a third is to avoid the *original remote cause.* A vermifuge is, in the first place, to be given. Though by no means ellegant, the following combination will be found very effectual: two drachms of finely pulverized radix spigelia, one drachm of finely pulverized seeds of the chenopodium, mixed with two ounces of a mixture of castor oil and the oil of turpentine, a portion of which is to be taken every two or three hours, until free action of the bowels ensues. The second indication is to be fulfilled by gentle tonics, cathartics, alteratives, &c. If borne, iron will be found extremely serviceable *now.* The third indication will be found more difficult of fulfilment than either of the preceding. The digestive system must be kept in good condition; to do which requires, however, not unfrequently more temperance and more dietetic restrictions than we can enforce our patients to observe. We are so constituted, that even slight, often repeated, violations of the laws of our being, subject us to inexpressible misery. And, unfortunately, the temptations to infract those laws are, to a comparatively uneducated and unrefined class of people—such as, I regret to say, too many of *us* are—stronger than the beautifully symmetrical laws of God. But it is the duty of every high-minded physician to do his might, to labor assiduously, not only to repair those breaches, the effects of the insulted laws of man's nature; but also to elevate him, both mentally and morally—thereby insuring to him the best possible condition of his physical frame.

